# Recycling, Remanufacturing and Applications of Semi-Long and Long Carbon Fibre from Waste Composites: A Review

**DOI:** 10.1007/s10443-025-10316-6

**Published:** 2025-03-11

**Authors:** Behzad Abdi, Yong Wang, Hugh Gong, Meini Su

**Affiliations:** 1 Department of Civil Engineering and Management, University of Manchester, Manchester, UK; 2 Department of Materials, University of Manchester, Manchester, UK

**Keywords:** Application, Carbon fibre, Mechanical properties, Recycling, Review

## Abstract

Carbon fibres can be reclaimed and processed to different forms as feed material to make remanufactured carbon fibre composites. Use of semi-long (25–100 mm) and long (> 100 mm) reclaimed carbon fibres in composites has the potential to enhance the overall mechanical performance of composites made from reclaimed carbon fibres. However, the present processes of recycling of carbon fibres lead to shortening of fibre length, surface degradation, alignment, which in turn, decrease the load bearing capacity and matrix bonding in the composites. To increase the structural performance and mechanical characteristics of reclaimed carbon fibres-based composites, possible pre-treatment methods to semi-long/long reclaimed carbon fibres should be explored. This paper presents a detailed review of various preparation and remanufacturing processes for semi-long/long reclaimed carbon fibres and evaluation of their performance and potential applications. It is found that among all the recycling methods, the Electrically driven Heterocatalytic Decomposition method can produce semi-long/long reclaimed carbon fibres with minimal damages. After reclaiming the carbon fibres, they must be opened and separated from the fluffy form for further processing; long staple carding is one of the mostly used methods for opening and producing randomly aligned mats and tapes. To enhance the performance of composites made from semi-long/long reclaimed carbon fibres, it is essential that fibres are aligned unidirectionally as much as possible. Friction spinning is found to be an efficient method to achieve high alignment of semi-long/long fibres. Furthermore, this paper advocates the use of advanced manufacturing techniques for fibre alignment and customization, which could result in improved repeatability, reduced variability, reduced material waste, and increased suitability for specific applications.

## Introduction

Composites are structures in which the polymer resins act as a matrix to hold fibres and provide load transfer and distribution to the structure as well as to the fibres thereby increasing the mechanical and durability properties of the structure [1]. Furthermore, resins also offer protection from the environment by preventing the infiltration of water, ultra violet light and other chemicals in the fibre leading to composite durability under various service conditions [1]. In recent decades, carbon fibre (CF) based composites have seen dramatic growth in usage in various sectors including aerospace, automotive, railways and renewable energy owing to their exceptional mechanical properties, lightweight, and good wear and fatigue resistance [2, 3]. In 2020, its market size was USD 3.7 billion, and by 2031 it's expected to be USD 8.9 billion [4]. However, virgin CFs are expensive (costing between USD 35 and USD 65 per kilogram [5]) and its production can be hazardous to human health [6] and energy extensive (i.e. about 183–286 MJ energy for producing 1 kg CFs) [7].

The large demand for CFs and its high cost attracts the composites industry to consider reclaiming CFs from end-of-life CFRP composites. Compared to manufacturing new CFs, recycling CFs from wastes such as composite production offcuts, end-of-life composite parts, and manufacturing defects, requires less energy and thus is much less expensive (~ USD 5.0 per kilogram [5, 8, 9]). More importantly, in many developed countries, recycling CFRP composites is the main solution to managing wastes because landfill or burning composite materials has been prohibited [10]. Due to European Directive 2000/53/EC on end-of-life vehicles, which requires reusing and recycling 85% (and further 95%) of vehicle parts, there is increasing interest in recycling for advanced material like CFRP. While the directive is aimed at vehicles [11]. The same issue and incentive apply to the automotive industry where it is anticipated that by 2030, about 6,000 to 8,000 aircrafts will reach the end of the service life [12]. Against this background, efficient methods for recycling and reusing of CFs is urgent [13–15]. Unfortunately, the CFs reclaimed from the existing recycling methods are discontinuous and fluffy; therefore, additional processing is necessary before using rCFs as the feed materials for remanufacturing. Unlike previous reviews that mainly review general methods of reusing rCFs, this review specifically focuses on some of the recycling systems and specific re-processing and remanufacturing techniques for semi-long/long rCFs derived from waste, and their possibilities for application in producing advanced composites [9, 16–19]. The review also examines the potential applications of these rCFs in the manufacturing of composites with higher performance. Furthermore, it discusses the main steps involved in the process of converting wastes into new composite products, in particular reusing semi-long/long rCFs.

This review paper is structured into five sections. Section 2 outlines the recycling techniques for both thermoset and thermoplastic composites. Section 3 covers the re-processing and re-manufacturing of semi-long/long length rCFs. Section 4 discusses potential applications of remanufactured CFRP composites. Section 5 summarises the key findings of this review.

## Recycling of Carbon Fibres from Composites

Depending on whether the composite is thermoplastic or thermoset, the most appropriate recycling methods will be different. This is because there are fundamental differences between thermoplastic and thermosetting resins, which would suit different recycling and remanufacturing techniques [20]. When thermosetting resins are cured, they undergo chemical reactions that produce a rigid, cross-linked structure. The irreversible curing process renders thermoset composites incapable of remoulding or reshaping, which poses considerable challenges in recycling. In contrast, thermoplastic resins are reversible, and it is much easier to remould and reshape thermoplastic composites. The distinct properties of thermoplastic and thermosetting resins play a fundamental role in determining whether CF composites are recyclable [21]. Although not directly in the scope of this paper, thermoplastic composites are well suited to in-situ repairs, damage healing, and remanufacturing [22]. Thus, the ability to repair thermoplastic composite components reduces the need to recycle thermoplastic composites.

**Fig. 1:**
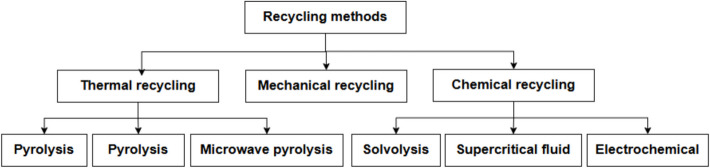
Methods of recycling CF from waste composites

The existing recycling methods for reclaiming CFs from wastes can be generally classified into three main categories—mechanical [23, 24], thermal [25, 26] and chemical/electrochemical [17–20] methods, as summarised in Fig. 1. In this section, the commonly used recycling methods for both thermoset and thermoplastic composites are discussed.

### Recycling from Thermoplastic Composites

Mechanical recycling is an efficient and relatively affordable process, whereby waste thermoplastic composites are disintegrated into fine powder or fibrous material of variable lengths with high resin content [27]. The rCFs obtained from this method can be used as fillers or reinforcements in various applications by employing techniques such as injection moulding and thermocompression moulding [28]. Mechanical recycling, however, damages the reclaimed fibres and reduces their residual mechanical properties to approximately 50 to 65% of the virgin CFs [29]. In addition, the quality of rCFs will be affected by contaminations from the residual matrix materials [30, 31].

**Fig. 2:**
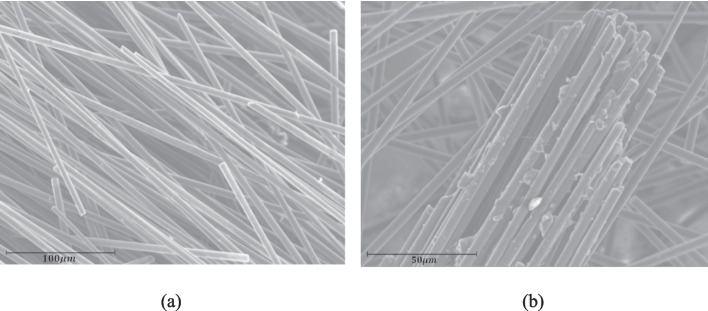
Surface quality of thermally recycled rCF, (**a**) clean surface, (**b**) surface with char [37]

A variety of thermal recycling methods can be used to recover high-quality composite fibres of thermoplastic composites while producing energy at the same time [32, 33]. In pyrolysis recycling, thermoplastic composites are decomposed in an oxygen-free atmosphere which leave the fibres intact [34, 35]. In this process, composites are placed in a reactor in the absence of air and heated to temperatures usually between 400°C and 700°C [36]. Due to high temperatures, the polymer matrix breaks down into volatiles, allowing the CFs to be recovered as the final solid residue. However, this process can result in some residual char like deposit on the fibres (Fig. 2b), degraded bonding interface and impurities which must be subjected to further surface treatment in order to restore the mechanical properties [37]. The gasification recycling method is like the pyrolysis recycling method, involving temperatures above 700°C. Gasification uses controlled oxygen or steam in comparison to the pyrolysis method where it operates in an absence of oxygen [38, 39]. A polymer matrix is converted into high-energy gaseous syngas (a mixture of hydrogen, carbon monoxide, and other gases), which is collected for energy production. The CFs are then separated from any char. Though pyrolysis and gasification processes generate syngas, gasification is primarily designed to produce a clean, gaseous fuel while pyrolysis also produces large amounts of liquid and solid co-products. Gasification is also considered to be more energy efficient per ton of waste than incineration because it can produce both heat and electricity. however, the high temperatures and an oxidative environment can result in significant damage to the recycled CFs. Thus, the focus of further development in thermal recycling is to improve reactor design and pyrolysis conditions to achieve the highest energy recovery, fibre quality, and process efficiency, in addition to surface treatments to remove any residual char and to improve bonding behaviour between rCFs and polymer.

CFs can also be recycled from waste thermoplastic composites by chemical recycling [4, 40]. Chemical recycling techniques include solvolysis and chemical depolymerization. The solvolysis process breaks down the matrix of composites using solvents [33]. In this process, the composite materials are soaked in a solvent to dissolve the polymer, followed by heating, and then recycling the CFs from the degraded polymer solution [35]. The physical and mechanical properties of rCFs such as surface quality, tensile strength, length and purity depend on temperature, pressure, and solvent type of the process [41]. The main advantage of solvolysis process is the ability to achieve specific degradation of the matrix while leaving the fibres unaffected, resulting in rCFs of high quality. Nevertheless, for this method to be economically and environmentally viable, it is essential to overcome challenges of solvent recovery and handling of the by-products. In depolymerization recycling, composites are treated with selected depolymerizing agent, and the specific chemicals are used to dissolve the polymer matrix into its monomers or oligomers [42, 43]. Afterwards, CFs are removed from the decomposed matrix [44]. The decomposed matrix can be reused. Therefore, this method has the advantage of recycling both the matrix and the fibres of composites, making it a closed-loop process. This method also results in high quality rCFs. However, chemical recycling poses its own set of challenges, including being an energy-intensive process and needing. specialized equipment and techniques, which can make scaling difficult. Moreover, compliance with regulations regarding the handling and disposal of chemicals adds to the complexity and cost of chemical recycling operations [45].

### Recycling from Thermoset Composites

High-performance composites normally use thermoset matrix, which poses major environmental concerns because the stable chemicals used in such composites may not decompose in the natural environment for thousands of years. Thermoplastics encounter the same issues, although the difficulties increase with thermosets because they cannot be melted, reshaped, or easily dissolved from the fibres after curing. As thermoset and thermoplastic composites require different recycling processes due to their distinct material properties, resulting in caried outcome for different purposes, this paper will mainly focus on overcoming the significant challenges of recycling thermoset composites. As with thermoplastic composites, thermoset composites can be recycled mechanically, thermally, and chemically [46, 47].

#### Mechanical Recycling

During the mechanical recycling techniques, the waste thermoset composites is broken down mechanically into powdered or fibrous materials (1 μm to 25 mm). It typically involves several steps, including crushing, grinding, and milling the waste composite to smaller particles. rCFs are then separated from the thermoset matrix by sieving, air classification, or electrostatic separation. Mechanically rCFs can be affected by the efficiency and quality of the recycling techniques, the waste composite’s quality, and the post-processing steps.

Mechanical recycling processes do not alter the chemical compositions of the fibres, but the surface characteristics and mechanical strengths of the fibres are degraded due to residual resin and mechanical wear. This preservation is achieved because the process operates at ambient temperature, thus eliminating any risk of oxidation or graphitization that typically occur at temperatures between 400°C to 500°C and above 1000°C, respectively [48]. Furthermore, mechanical recycling is generally more cost-effective compared to other methods because it requires less energy input and can be carried out using relatively simpler equipment and process. Additionally, mechanical recycling produces fewer harmful emissions compared to thermal recycling. However, this process may introduce impurities and contaminants which originate from the thermoset matrix or the recycling process itself into the rCFs, which negatively impact the properties and quality of the rCFs. All these will result in poor mechanical and structural performance of the composites made by rCFs. Therefore, mechanically rCFs are typically employed in non-structural applications including lightly loaded automotive parts, sporting goods, and consumer products. It is also reported that scaling up mechanical recycling processes to handle large volumes of waste thermoset composites can be challenging [48].

#### Thermal Recycling

Thermal recycling utilizes high temperatures to decompose the thermoset polymer in waste composites and reclaim CFs [46]. This process typically includes shredding and size reduction, followed by heating and pyrolysis and CF recovery. Unlike mechanical recycling, thermally recycled rCFs allow the recycling of relatively longer rCF regardless of the possible reduction in length after the shredding step that often precedes thermal treatment. However, the high temperature thermal recycling process degrades the CFs which usually can occur in two mechanisms including oxidation (400°C to 500°C) and graphitization (over 1000°C) [48], thus reducing their mechanical strength and stiffness compared to the virgin CFs. While thermal recycling is effective in breaking down the thermoset composites, this process comes with several side-effects including microcracks, voids, oxidation, and residual impurities, which may compromise the mechanical properties and structural integrity of the rCFs [48]. To mitigate these defects, several strategies can be employed during thermal recycling. Methods include controlling the atmosphere environment, optimizing temperature profiles and heating rates and incorporating additives or modifiers [49]. By conducting the recycling process under an inert gas atmosphere, such as nitrogen or argon, oxidation reactions can be prevented [48]. Vacuum environments can also be used to completely remove oxygen, preventing oxidative degradation during the thermal recycling process [46]. Adjusting the heating rate and dwell time can also help optimize the thermal degradation process, thereby minimizing microcracks and voids [50]. In addition, post-treatment techniques such as annealing, or surface modification can be used to minimize the defects introduced during thermal recycling [14]. But all these treatments incur penalties in time, cost and environmental impacts.

#### Chemical Recycling

Chemical recycling is to decompose the thermoset matrix of waste composites to reclaim its constituent monomers and CFs through various chemical processes including depolymerization, solvent extraction, purification and fibre regeneration [48]. The depolymerization step involves breaking the chemical links of the polymer network to convert the thermoset resin into smaller and more manageable molecules. Once the thermoset matrix is depolymerized, solvents can be used to selectively extract rCFs from the mixture. The selection of solvent is usually determined by the type of thermoset resin and its compatibility with the solvent. The extracted CFs are then subjected to purification processes to remove any remaining impurities, such as residual resin or contaminants to increase the overall mechanical and physical properties of the rCFs. The rCFs can undergo additional treatments, such as thermal or chemical treatments, to improve their mechanical properties and to restore their performances as closely as possible to those of the virgin CFs. The properties of rCFs reclaimed by chemical method are affected by several factors, including the quality of the waste thermoset composites, the efficiency of the depolymerization and purification processes, and the treatments applied to the fibres. However, the costs associated with depolymerization, solvent extraction, purification, and fibre regeneration can be very high. The technology of chemical recycling is still in its infancy, and scaling up the procedures to deal with large volumes of waste thermoset composites represents a significant challenge. Traditional chemical recycling processes involve the use of solvents and substances which leads to environmental pollution; however, recent developments of advanced chemical recycling methods greatly reduce environmental impacts [51, 52].

#### Electrically Driven Heterocatalytic Decomposition Method

The recently developed Electrically Driven Heterocatalytic Decomposition (EHD) method is a promising technique for recycling CFs from waste thermoset composites [29]. The EHD method applies an electric current to the thermoset composite in a solvent catalyst system (de-anodized water, NaCl and Potassium hydroxide (KOH)), which induces catalytic decomposition of the resin. The catalyst, typically a metal or metal oxide, facilitates the breakdown of the resin into volatile products, leaving behind rCFs. The decomposition process occurs at room temperatures, reducing the risk of thermal degradation of rCFs [29]. Therefore, the EHD process can achieve high retention of rCF mechanical properties, ranging from 85 to 95%. This is because this process avoids thermal degradation, impurity or mechanical cutting associated with the aforementioned other processes. It is also noted that another notable advantage of the EHD method is its simplicity [29].

Table 1 summarizes the main features of the different recycling techniques and the key properties of the rCFs.

**Table 1:** Different features of recycling methods and key properties of the rCF

Method	Mechanical	Thermal	Chemical	EHD
Mechanical properties	20–35% of virgin fibre [60–62]	80–90% of virgin fibre [63, 64]	90–95% of virgin fibre [29, 65, 66]	90–95% of virgin fibre [29, 65, 66]
Fibre form	Powders/Fluffy [60, 67]	Fluffy [68–70]	Fluffy [68–70]	Fluffy
Surface quality	Coarse [62]	Clean surface quality [47, 71]	Clean surface quality [65, 66]	Clean surface quality
Fibre length	Short (1 μm-25 mm) [62]	Various fibre length [60, 72]	Various fibre length [68–70]	Various fibre length
Interfacial bonding strength	Low (< 50MPa)	Good (50-150MPa)	High (> 200MPa)	High(> 200MPa)
Fibre recovery rate (%)	Reduced due to fragmentation	Up to 95–98%	Up to 93% retention of performance	High-quality fibres with minimal damage
Cost (USD/kg) (equipment, energy, and consumables)	1–5	20–30	25–50	10–15
Environmental impact	Energy consumption: 0.27–2.03 MJ/kg [73] Greenhouse Gas Emissions: 378 kg CO2eq./t [74]	Energy consumption: 3–30 MJ/kg [73] Greenhouse Gas Emissions: 1.52 kg CO2eq./t [75]	Energy consumption: 19.2-91MJ/kg [73] Greenhouse Gas Emissions: 1.52 kg CO2eq./t [75]	Energy consumption: less than 15MJ/kg [76] Environmentally friendly method
Complexity (scale 1–5)	1	3	4	2
Scalability (scale 1–5)	4	5	3	4

After the recycling of fibres, fibre surface coating or sizing plays an important role in the manufacturing of composites [53, 54]. Surface coating or sizing becomes more important for rCFs due to the possibility of residuals present on the fibres and weak interfacial bonding between rCFs and new matrix. In surface coating or sizing, the fibres are coated or sized with a thin layer of compatible material such as epoxy-based sizing materials and silane-based sizing agents to enhance the compatibility and adhesion of the fibres to the new matrix [55]. Thus, sizing serves as a bridge between the fibres and the matrix to provide the required interfacial bonding and to enable the materials to work compositely [56].

The sizing materials must ensure chemical resistance, thermal stability, and strong bonding within the composites. The selection of an appropriate sizing depends on the specific application requirements of the composite fabric and the type of resin used in the composite matrix. In addition to the selection of sizing materials, the method of application also needs careful consideration. Methods of application include Dip coating [57], intense pulsed light (IPL) [58], under which it is possible to control the application process precisely, resulting in uniform sizing coverage; irradiation [53], which alters the surface energy of the fibres; and plasma surface treatment [59] which is an environmentally friendly and versatile method capable of tailoring surface chemistry to optimize interfacial bonding in composite materials.

Among these different methods, the dip coating method is highly suitable for applying sizing materials onto discontinuous rCFs, due to its effectiveness in ensuring uniform coverage and the need for only a thin layer of sizing material on the fibre surfaces [77]. By immersing the fibres into a solution containing the sizing material, dip coating allows for thorough coverage of the fibre surfaces, ensuring consistent coating thickness and excellent penetration of the sizing material into the fibre bundles.

## Re-Processing and Re-Manufacturing

**Fig. 3:**
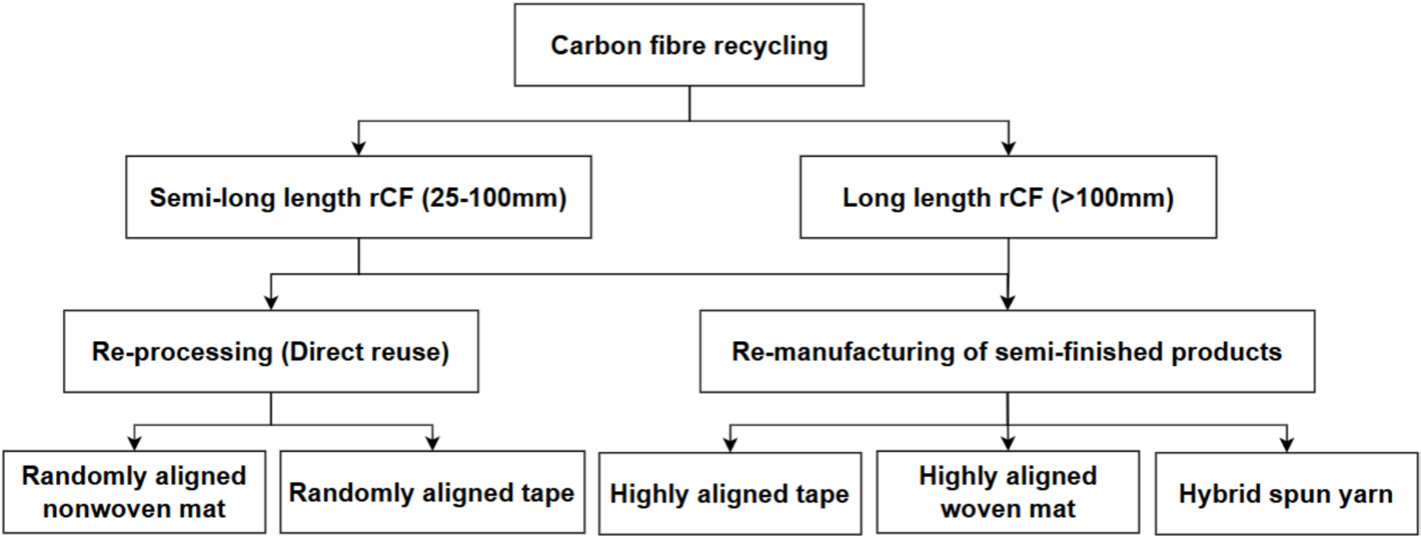
Overview of semi-products derived from rCFs through re-processing and re-manufacturing methods

The raw rCFs must be re-processed (direct reuse) and re-manufactured (semi-product) to achieve the potential of the fibres. In particular, since the focus of this paper is on using semi-long (25–100 mm) for applications requiring moderate reinforcement and flexibility in orientation within the matrix and long (> 100 mm) rCFs for enhancing load-bearing capacity of composites, this section assesses the suitability of various re-processing and re-manufacturing processes to semi-long and long rCFs, i.e. converting from a fluffy form to the desirable forms, as shown in Fig. 3. The approaches include the direct reuse in the form of randomly aligned mat and tapes, as well as re-manufacturing into highly aligned tape, highly aligned woven mat (fibres precisely arranged in a fabric to provide strength and stiffness in given directions) or continuous yarn.

### Re-processing of semi-long/long rCFs for direct reuse

**Fig. 4:**
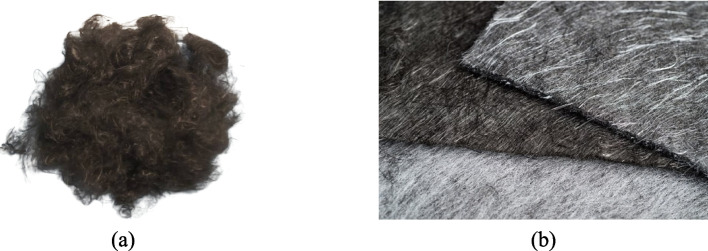
(**a**) Discontinuous and fluffy rCFs, (**b**) randomly aligned mats [80]

The use of semi-long and long rCFs in new composites depends on the following key parameters: fibre length and diameter, mechanical strength and the damage rate [78, 79]. In their raw form, semi-long and long rCFs are discontinuous and fluffy (Fig. 4a), which means they must be reprocessed to be converted into more useful forms such as randomly aligned mats or tapes (Fig. 4b). To accomplish the above, air-lay process, modified carding process, and wet-lay process may be used.

**Fig. 5:**
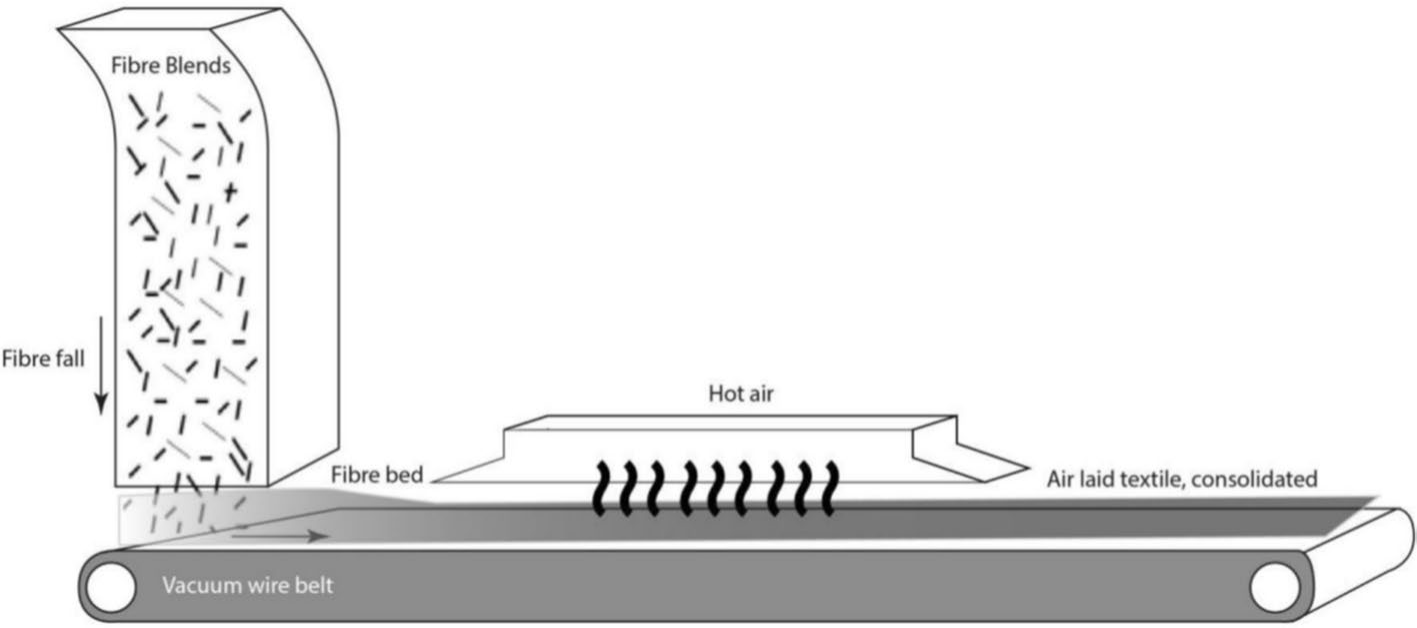
Schematic of air-laid process for the production of randomly aligned mat [81]

The air-laid process, as sketched in Fig. 5, is ideal for producing mats with varying thickness and density [8]. The main challenge in the air-laid process is to achieve a consistent dispersion of fibres throughout the material, ensuring no clumps or gaps ((uniformity) and minimizing variations in density and properties across the final product. rCFs used in the air-laid process should possess the required characteristics to ensure successful and efficient production [8]. In general, shorter fibres (3–10 mm) will produce a softer texture due to increased fibre entanglement and improved interlocking within the material, while longer fibres (10–20 mm) can enhance strength and integrity since they tend to interlock more effectively, producing a material that is more robust and cohesive.

The recycled fibres should be opened sufficiently because overly compacted or compressed fibres can cause difficulties in carding and reduce efficiency. There are two main types of carding processes, long staple carding and short staple carding. In long staple carding, multiple rollers are used in conjunction with a large cylinder to open, straighten and align fibres for uniform webs [82, 83]. Short staple carding opens and refines fibre alignment using a series of flats with card clothing in conjunction with the large cylinder [84, 85]. As the fibres pass through the cylinder carding surface, they are carded and blended, creating a web of fibres with a broadly machine direction orientation. Short staple carding is more compact and more intensive in fibre opening, but less versatile in terms of the fibres that can be processed. long staple carding machines are typically less efficient in fibre opening and bulkier than short staple carding processes but are capable of higher productivity [86]. Although long staple carding machines do not offer the ability of fibre cleaning, clean rCFs with the length of 10–250 mm can be used in the long staple carding process. For processing semi-long and long rCFs [84], the long staple carding method is thus more appropriate. In this process, the fibres are fed between two rollers covered with fine wire teeth, which helps to align and straighten the fibres [87]. The fibres are then transferred to a clearer, which further aligns the fibres. The method allows for better control and alignment of the fibres, resulting in a more consistent and uniform web [88]. In addition, it prevents fibre breakage and ensures that longer fibres are processed without excessive damage.

**Fig. 6:**
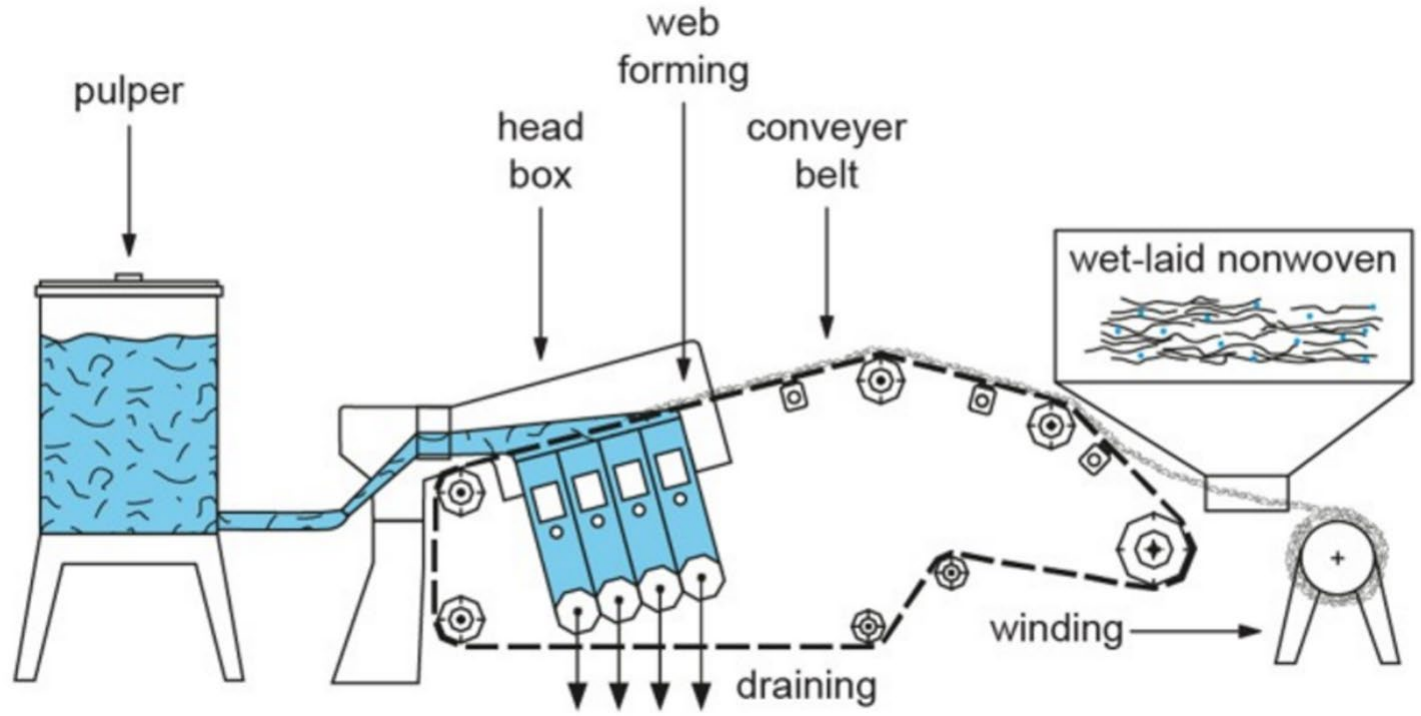
Schematic of wet-laid process for the production of randomly aligned mat [90]

The wet-laid process as illustrated in Fig. 6 is most suitable for producing mats with uniform fibre orientation and high mechanical strength [89]. The fibre length can be altered according to the applications and the intended products. However, the wet-laid process presents a challenge in achieving homogeneous dispersal of the rCFs in the liquid medium because the distribution and concentration of fibres within the slurry may be non-uniform, resulting in inconsistent mat properties. Furthermore, wet laying involves the use of a liquid medium, which can result in high moisture levels in the mat.

Table 2 compares the performance of the aforementioned three processes to produce rCFs for direct use. Wet lay, despite its drawbacks, appears to be the most ideal method, given its ability to handle a variety of fibres and the qualities of the resulting rCFs.

**Table 2:** Comparison of the main features of air-laid, wet-laid and carding processes to generate randomly aligned mates for direct reuse of rCFs

Process	Fibre conditions	Performance	Advantages	Disadvantages
Air-Laid [8, 92, 93]	• Lengths: 10–100 mm • Diameter: 5–50 µm • Minimal cleaning required	• Production speed: 50–100 m/min • Energy consumption: ~ 10 kWh/kg of material • High throughput due to simplicity in processing lightweight materials	• Porosity: 60–80% • Fibre damage: < 10% • Minimal equipment complexity • Porosity: 60–80% • Fibre damage: < 10%	• Tensile strength of the fibrous mat produced: < 15 MPa • Limited product thickness (< 5 mm • High capital investment for specialized equipment
Carding [84, 85, 94]	• Lengths: 10–150 mm • Diameter: 5–50 µm • Requires cleaning to remove contaminants	• Production speed: 30–70 m/min • Energy consumption: ~ 20 kWh/kg • Suitable for medium to heavy mats	• Tensile strength of the fibrous mat produced: 15–25 MPa • Thickness control: ± 5% • High adaptability to various fibre types and blends	• Lower production speed compared to air laid • High maintenance costs (~ 20% of equipment cost annually) • May struggle with very short fibres, resulting in material waste
Wet-Laid [92, 95]	• Lengths: 10–250 mm • Diameter: 5–50 µm • Requires extensive cleaning	• Production speed: 20–50 m/min • Energy consumption: ~ 30 kWh/kg • Superior mechanical properties for high-performance applications	• Tensile strength of the fibrous mat produced: 20–35 MPa • Fibre dispersion uniformity: ~ 90% • Versatility in product thickness and adaptability to demanding applications	• High water usage (~ 50–100 L/kg of material • Longer drying and curing times (24–48 h) • Increased residue generation due to water-intensive processing

**Fig. 7:**
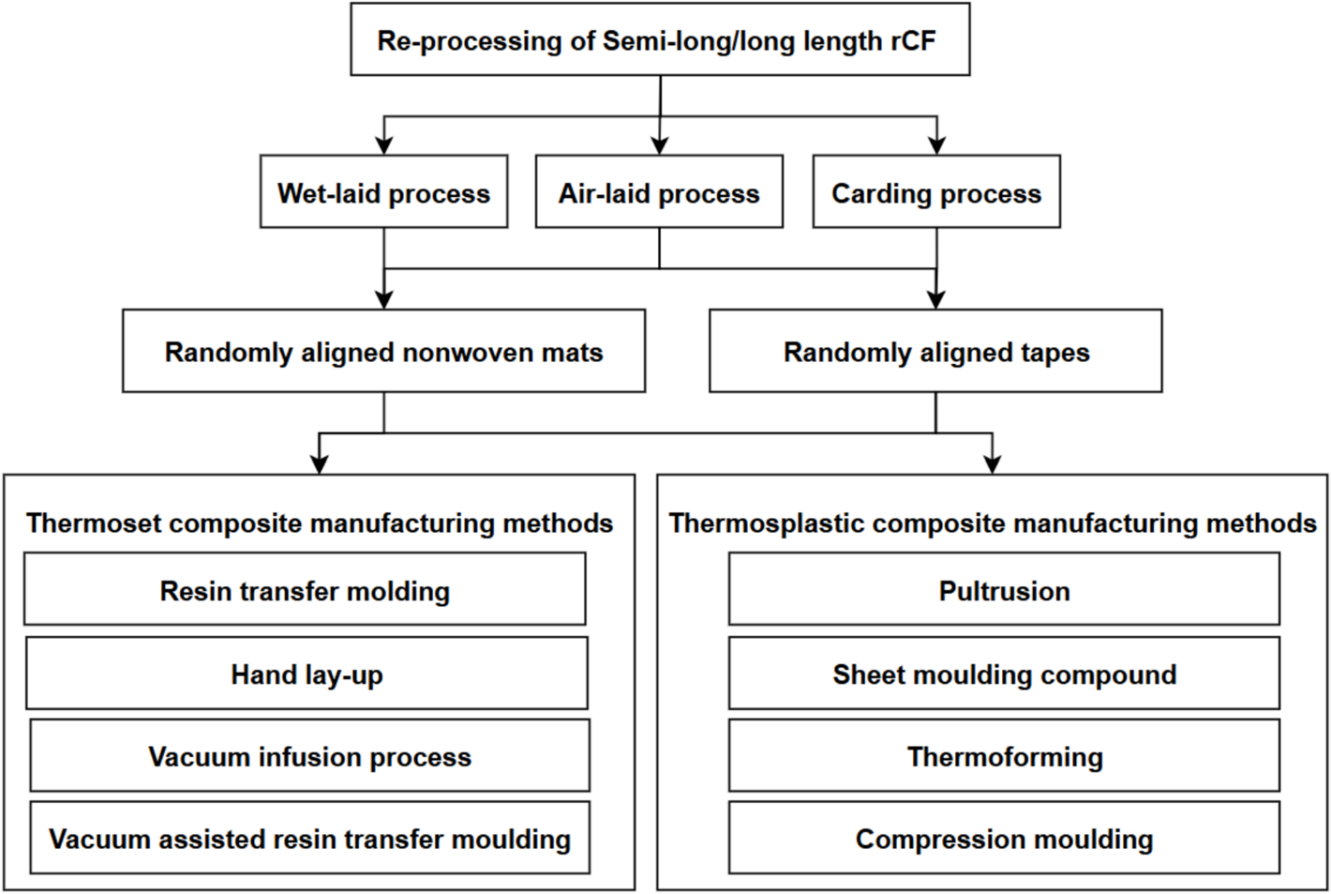
Re-processing of semi-long/long rCFs for direct reuse in composite manufacturing [96, 97]

Following re-processing, rCFs can be re-used in composites in a variety of ways, as illustrated in Fig. 7. In contrast, when semi-long or long reprocessed rCFs are directly incorporated into these composites, their mechanical properties are reduced, typically by 30%, due to insufficient fibre alignment during manufacturing. Furthermore, in the manufacturing processes for direct use of semi-long/ long rCFs, high pressure is used in compression moulding and automated tape placement processes while vacuum pressure is employed in the vacuum infusion process. In addition, the orientation of the fibres within the mat or tape may change, resulting in localized blockages of the flow channels and formation of high void content [91]. Because of the above disadvantages, direct reuse of semi-long or long rCFs (reprocessing) is seldom pursued in high quality application of composites, remanufacturing is necessary.

### Re-Manufacturing Using Semi-Long/Long rCFs

The remanufacturing methods can be dry or wet, and a number of remanufacturing methods are available, including carding and drawing [98], electric field method [99], glycerine method [100], centrifugal alignment [101], and the High Performance-Discontinuous Fibre (HiPerDiF) method [102].

The glycerine alignment technique is a relatively simple and inexpensive wet approach [100]. In this process, the glycerine solution acts as a lubricant, allowing the fibres to move and slide easily within the solution to align in the direction of the flow. This method can be used to align fibres in a variety of directions, but the fibres must be clean and of sufficient length (25–75 mm) to achieve effective alignment. There are a few factors that influence the alignment of the fibres, including the type, length, the concentration of glycerine, and the method used to place them on the slide. The method is suitable for alignment of fibres whose lengths are in the range of 25–75 mm, however, if the method is used for short fibres (< 25 mm) or long fibres (> 75 mm) the composite will be weak because of the fibre’s poor alignment. Furthermore, the fibres should have similar diameters to ensure uniform alignment and distribution. The glycerine method also has some limitations. The process can be labour-intensive and complex, requiring specialized equipment and skilled operators, which may increase the overall manufacturing cost. Additionally, it may be difficult to align fibres in a precise and uniform manner since it may be difficult to make all fibres maintain a consistent orientation throughout the entire composite material. Furthermore, glycerine can leave a residue on the aligned fibres, which can adversely affect the properties of the composites [6].

When using centrifugal force to align and separate rCFs [101], the rCFs are placed in a spinning chamber where they are rotated at high speeds. The fibres are split according to their lengths and aligned in the direction of the spinning due to the centrifugal force of spinning. This method is most effective when fibres are long (25–75 mm). In centrifugal alignment, the alignment angle can only be partially controlled, and it may not be possible to precisely control the orientation of the fibres to meet some specific requirements. The batch size for centrifugal alignment may also be limited by the size and capacity of the equipment, which can limit its scalability for large-scale production.

Another manufacturing process is the HiPerDiF which is an acronym for High-Performance Discontinuous Fibre that is a method of making high-performance composites applying discontinuous fibres, from recycled carbon or glass fibres [102]. It can accommodate fibre sizes from about 3 to 12 mm giving way for their controlled orientation which improves the mechanical characteristics such as flexural and tensile strength [103]. This method is suitable for all types of fibre and has very high advantages in the areas of sustainability and affordability since it incorporates recycled fibres [103, 104]. Nonetheless, HiPerDiF involves stringent procedure and, apparatus, which maybe expensive and sensitive and cannot work with fibres that are too short and specific thermoplastic resins. While it can yield composites with properties closer to the composites made from continuous fibre, achieving uniform fibre distribution and fabrication of materials with varying fibre lengths makes it difficult [103].

**Fig. 8:**
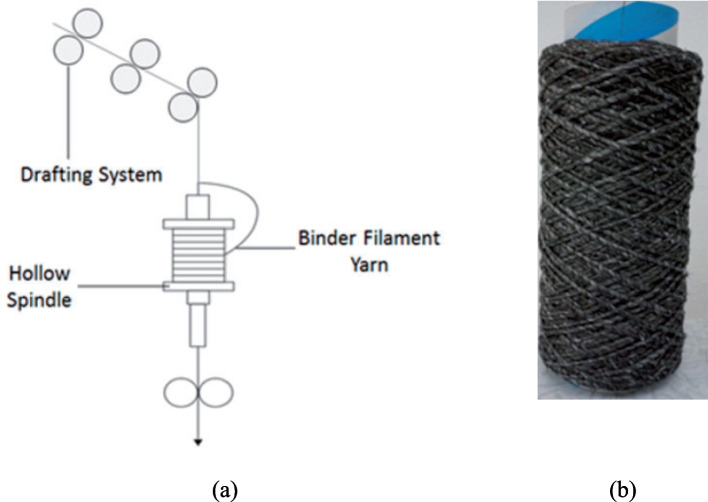
(**a**) A diagram of the wrap spinning process, (**b**) a rCF based hybrid spun yarn [109]

**Fig. 9:**
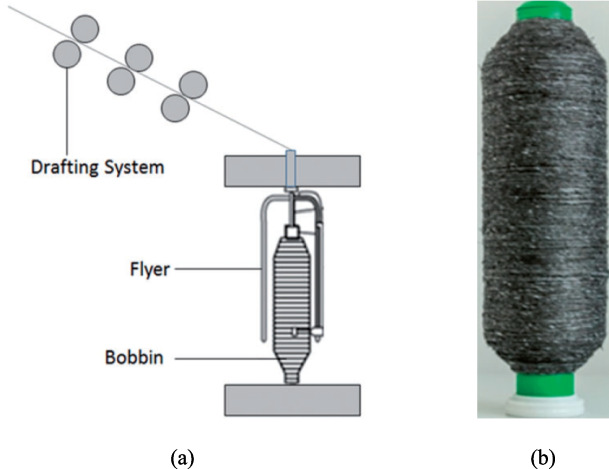
(**a**) A diagram of flyer spinning method, (**b**) a rCF based hybrid yarn [106]

**Fig. 10:**
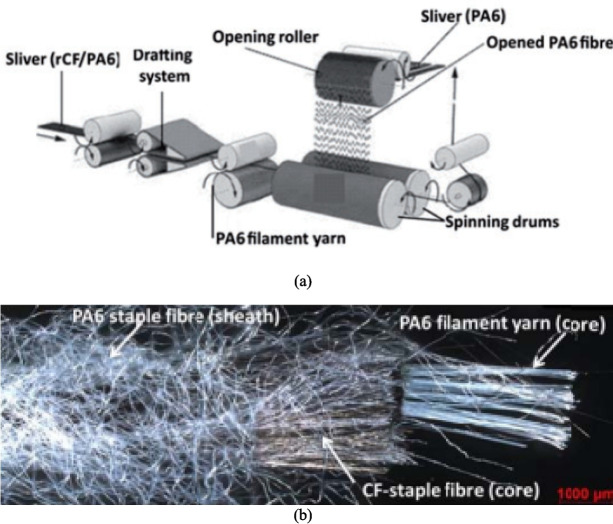
(**a**) A diagram of friction spinning method, (**b**) a rCF and PA6 staple fibre-based hybrid spun yarn [107, 108]

Yarn spinning methods include wrap spinning, flyer spinning and friction spinning. All of them are widely used dry alignment methods to convert discontinuous fibres into yarns by aligning and twisting them together [19, 105], as shown in Figs. 8–10, respectively. Wrap spinning produces uniform and highly aligned hybrid spun yarns, making it ideal for the fabrication of composites made from rCFs (Fig. 8) [19]. Flyer spinning is a versatile process that produces uniform hybrid spun yarns with a range of densities, ranging from 200 to 3500 Tex (Fig. 9) [106]. Friction spinning is capable of producing hybrid spun yarns made from various types of fibres, including semi-long rCFs [107, 108]. As illustrated in Fig. 10, this method involves feeding the fibres to the nip point of two spinning drums under vacuum condition, where they are twisted and compressed into a high-strength core-sheath yarn. The main advantage of friction spinning is its ability to process fibre materials containing large variations in fibre length. This makes it more suitable for converting recycled discontinuous fibres to continuous yarns because recycling processes usually produce fibre materials with a mixture of length. Another advantage of friction spinning is its ability to produce hybrid yarns with a core/sheath structure whereby a core can be used to enhance the mechanical performance of the final yarn.

Based on theoretical analysis, some of the key parameters affecting the effectiveness of the friction spinning process include:

- **Fibre preparation:** Fiber preparation for spinning rCF is essential for achieving high-quality yarn with desirable properties. This process involves thorough cleaning to remove contaminants, carding and drafting to align the short, discontinuous fibres, and blending with other fibres if needed to enhance the strength and consistency of the final yarn [110, 111].
- **Friction drum speed:** The speed at which the friction drum rotates during friction spinning affects yarn quality. Higher friction drum speeds produce more twist and a denser yarn with improved strength, but excessively high friction drum speeds can cause instability and weaken the yarn [112, 113].
- **Fibre length:** Long (typically greater than 60 mm) are desirable for friction spinning as they produce strong yarns due to better binding opportunities [85]. In rCF textile processing, sufficient fibre length is an important factor to achieve adequate cohesion and alignment, which improves the overall mechanical characteristics of the final yarns. However, using only long fibres may not always be practical or cost-effective, particularly for some fibre types or blends [105, 114, 115].
- **Air suction pressure:** Air suction pressure or vacuum pressure is applied to the fibres from within the friction drums to generate the friction force needed to twist the yarn. Studies have shown that higher vacuum pressures can increase the tenacity and result in a stronger and more compact yarn [116–118].
- **Core delivery speed:** In production of rCF based yarn employing friction spinning, core delivery speed plays a role in determining the core-sheath blend ratio by adjusting the proportion of core material relative to the sheath. Friction spinning converts fibres into yarn using the combined action of twin high-speed rotating drums instead of the standard spinning approaches. As the sheath fibre feed rate along with spinning speed determines sheath fibre twist and yarn density so the core delivery speed stands alongside to enable yarn development through distinctive drum friction forces [119–121].

Table 3 specifies a comparison of the different remanufacturing processes for rCFs, and Table 4 compares the key characteristics of the resulting rCFs after these remanufacturing processes.

**Table 3:** Comparison of the main features of different remanufacturing processes for semi-long and long length rCFs

Method	Fibre conditions	Advantages	Disadvantages
Carding	• Various lengths • Various diameters • Various fibres • Clean	• High production speed • Versatile for different fibres • Established industrial process	• Not precise alignment • Low alignment control • Limited to non-woven mats and yarns
Glycerine	• Medium lengths, Consistent diameter • Clean	• Can achieve controlled alignment • Low mechanical stress on fibres • Relatively simple setup and process	• Alignment may not be permanent • Requires additional steps to solidify alignment • Limited to certain fibre types and applications
Centrifugal alignment	• Semi-long and long lengths • Consistent diameter • Clean	• Can achieve high alignment degrees • Suitable for high-performance composites	• Limited to long fibres • High mechanical stress on fibres • Expensive equipment and setup
Friction Spinning	• Semi-long and long lengths • Various diameters • Clean	• High alignment efficiency • Continuous production process • Can produce yarns with different cross-sectional shapes or alignments for specific applications	• Requires precise control and processing • May not be ideal for very short or highly delicate fibres • Moderate setup and equipment costs
HiPerDiF	• Various lengths • Consistent diameter • Clean	• High alignment precision • Can handle various fibre types • Scalable for industrial production	• High initial setup cost • Requires specialized equipment • Complex operation and maintenance

**Table 4:** Key characteristics of rCFs after different remanufacturing processes

Method	Fibre orientation	Alignment control (%)	Fibre damage (%)	Processing speed (m/min)	Equipment cost (USD)
Carding [85, 94]	± 30° (Random)	< 20%	20–30%	50–100	50,000–100,000
Glycerine [124, 125]	± 10° (Predominantly Aligned)	70–90%	5–10%	30–50	10,000–30,000
Centrifugal Alignment [126, 127]	± 10° (Predominantly Aligned)	70–90%	5–10%	30–50	800,000–120,000
Friction Spinning [113, 128]	± 5° (Predominantly Aligned)	90–95%	10–20%	50–250	30,000–70,000
HiPerDiF [103, 104]	Highly Aligned	> 80%	< 10%	Not specified	Not specified

**Fig. 11:**
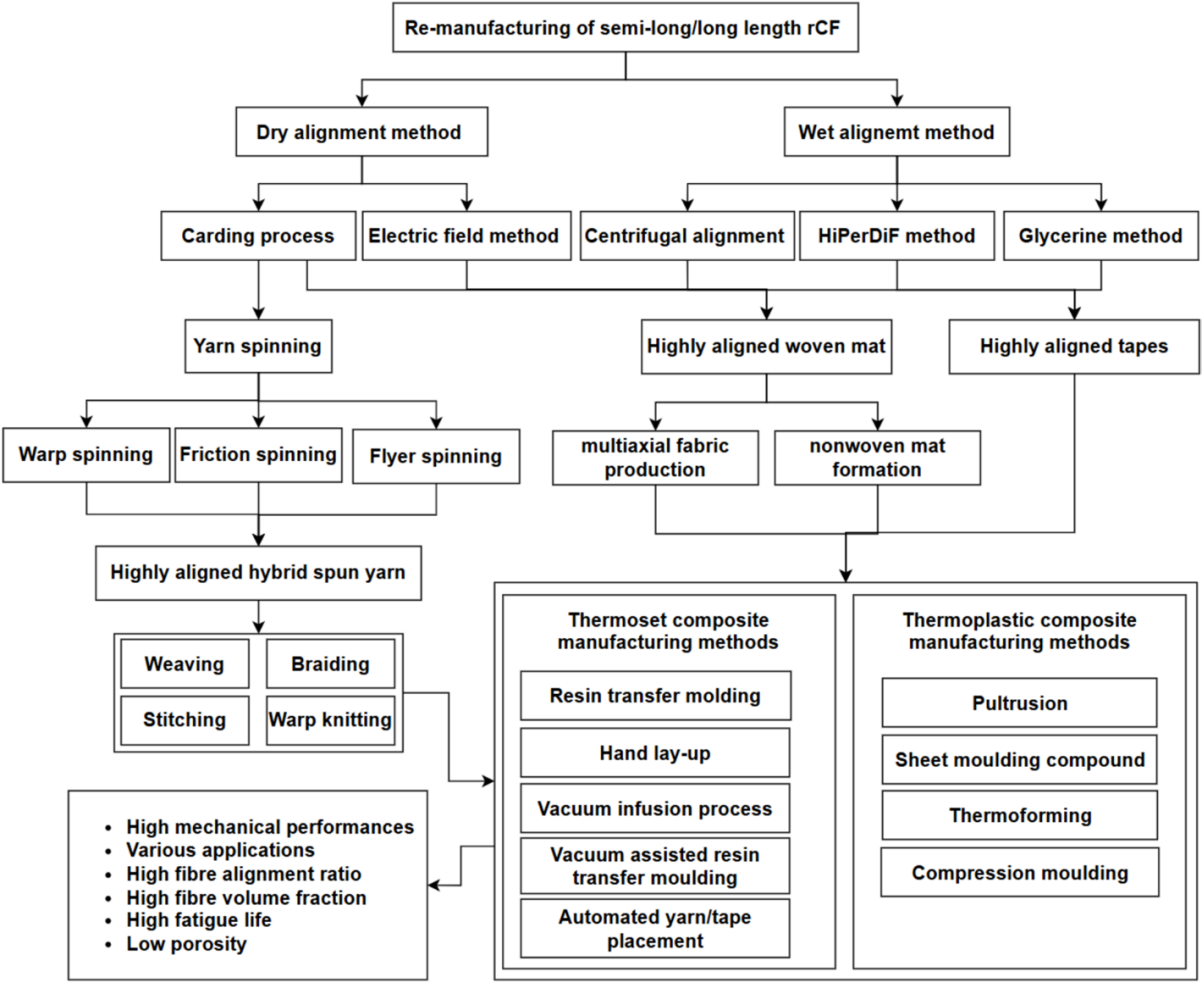
Methods of re-manufacturing and application of semi-long/long length rCFs for production of high-performance composites

By focusing on technical performances while assuming that problems of setup and equipment cost can be solved through investment and training, friction spinning would be the most preferred choice for remanufacturing with rCFs due to its efficient processing speed, high alignment ratio and moderate equipment cost. The simple setup and moderate costs of friction spinning make it an excellent solution compared to costly centrifugal alignment and difficult HiPerDiF methods. Its use with different fibre types at different configurations gives it broad applications. For industrial use friction spinning gives better alignment results than expensive centrifugal alignment or more complex HiPerDiF but less expensive. Its precise alignment technology boosts composite strength, and stiffness plus lets the process run steadily on a large scale. Carding and glycerine production remain among the simplest approaches but yield poor structural alignment which makes them unsuitable for advanced composites. With friction spinning method, semi-long/long fibres can be aligned and processed, improving their mechanical properties and overall performance. By controlling fibre orientation precisely and producing continuous yarns, it ensures greater consistency, strength, and suitability for high-performance applications. The yarns made by rCFs can be used in high performing composites using a variety of application methods, as outlined in Fig. 11.

## Applications of Semi-Long/Long Length rCF

As has been presented in detail in this paper, being able to reuse semi-long and long CFs bring about a lot of advantages such as reduced cost, high retention of mechanical properties. For example, in the aerospace industry, where lightweight materials are crucial for fuel efficiency and performance, the use of semi-long and long rCFs could significantly reduce costs while retaining high mechanical properties. Similarly, in the automotive sector, incorporating these recycled fibres into components such as chassis or body panels could offer substantial weight savings without compromising strength.

Semi-long and long rCFs are at present used in selective industrial applications because of the problems of quality control, fibre alignment, and strength [122]. In the process of recycling, mechanical properties of rCFs may decrease, and this variability is undesirable and takes time when it comes to quality control. Perfect fibre alignment is however challenging and compromises the load bearing capability of composites; poor interface compatibility arising from inability to modify fibre surface to interact properly with matrix material, reduces the overall strength of composites. Furthermore, equipment not designed specifically for handling rCF can cause significant breakage furthering issues with processability and cost [123]. Long rCFs (> 100 mm) have less flexibility, which means they cannot easily be bent to form components with intricate shapes and designs, reducing compatibility with pathways that have an elaborate design Moreover, costs for quality checks and special treatments of the reclaimed fibres are added to cost, which may even exceed the costs for virgin CFs. It creates an economic drawdown, especially if using rCFs in high performance fields, for instance aerospace or automotive.

Nevertheless, with further research, significant strides can be made in improving the quality of rCFs and enhancing the remanufacturing process, thereby increasing the industrial application of semi-long and long rCFs. Detailed research areas include refining sizing materials for better adhesion and compatibility with rCFs, thereby improving their dispersion and bonding within composite matrices [77]. Additionally, investigating novel remanufacturing processes, such as advanced sorting and purification techniques, could help mitigate challenges associated with fibre variability and contamination [129]. Furthermore, optimizing processing parameters during moulding or additive manufacturing, could improve the mechanical characteristic and surface finish of composites. Additionally, studying the lifecycle impacts of incorporating rCFs into various industries and developing strategies for end-of-life recycling and reuse could further bolster the sustainability and widespread adoption of semi-long and long rCFs in industrial applications [130].

Through comprehensive research efforts in these areas, a significant step change can be achieved in unlocking the full potential of semi-long/long rCFs and revolutionizing their industrial utilization [131, 132]. To enable this, this section presents a review on conventional and advanced composite manufacturing methods using rCFs, to suggest plausible methods of manufacturing for applications of semi-long/long rCFs.

### Application and Performance of rCF-based Composites Made by Conventional Manufacturing Methods

Using rCFs in randomly aligned non-woven mat or tape forms in composites exhibits high drawability and formability, achieving similar mechanical and structural performances to virgin CFs in the same form. Nevertheless, the overall performance is low due to limitation in fibre length, dispersion, matrix compatibility, manufacturing parameters and application specificity [133–135].

To achieve high performance of composites using rCFs, rCFs should be unidirectionally aligned. For instance, a unidirectionally rCF composite can achieve a stiffness of 115 GPa [78]. Additionally, highly aligned woven or braided configurations are preferred for applications that require greater impact resistance or flexibility.

Liu et al. [124] reported that using a lower moulding pressure in an autoclave (at 120 °C, 7 bar pressure), fibre volume fraction ratio of randomly aligned rCF-based composites can reach 46%, resulting in the rCF composites achieving a tensile strength of 826 MPa, which is almost comparable to that made from virgin CFs which typically exhibit tensile strengths exceeding 1000 MPa, depending on the virgin CF type and sizing used. In this study [124], rCF-based composites are moulded at lower moulding pressures in an autoclave due to their unique characteristics. The lower moulding pressure prevents excessive compaction, preserving fibre integrity and alignment and minimizes fibre breakage. This technique facilitates a better preservation of rCFs, as well as their arrangement within the composite. Faure et al. [78] utilized long rCFs (up to 250 mm) in the production of unidirectional and bidirectional composites. They demonstrated that the fibre volume fraction ratio and tensile strength of unidirectional and bidirectional composites can reach 60%, 677 MPa and 50%, 400 MPa respectively. A combination of unidirectional and woven rCFs can be utilized to manufacture composites with improved mechanical characteristic in multiple directions. The composite resulting from the combination of unidirectional and woven rCFs can achieve superior strength and stiffness in one direction while also having good strength in other directions due to the bidirectional nature of woven fabrics [136]. Combining these properties allows for enhanced mechanical properties in multiple directions, making it ideal for applications with specific stiffness and strength in various directions required.

However, the incorporation of long fibres, especially up to 250 mm, can complicate the manufacturing process due to handling challenges, such as fibre entanglement and uneven distribution. Furthermore, obtaining consistent mechanical properties requires meticulous optimization and quality control measures. The proper orientation of fibre necessitates the use of specialized alignment systems, more processing steps, and more demanding quality assurance add further cost. Impregnation techniques of resin matrix variability are also necessary, increased testing, and defect mitigation for equally increasing production costs and time. Additionally, although the combination of different fibre orientations may enhance mechanical properties in multiple directions, this strategy would not be suitable if the performance demand in one direction of the composite is particularly strong compared to others.

Composites made from hybrid spun yarns using semi-long and long length rCFs have demonstrated great versatility and usefulness. The materials can be used in various manufacturing processes, including weaving, knitting, and braiding, to arrange yarns into semi-products for thermoset or thermoplastic composite manufacturing with specific geometries and properties for various uses, such as those in the aerospace and automobile sectors. The production of thermoplastic composites based on hybrid yarns has made good progress [107, 137]. Further attempts are being made to produce yarns for thermoset composites. For example, Hasan et al. [138] have used friction spinning process to develop rCF based hybrid spun yarn with ≥ 90% rCF content by weight for production of thermoset composites. Hassan et al. [138] reported that employing a heat treatment process before the production of composites enhances epoxy matrix impregnation and increase both Young modulus and tensile strength of composites to 86 ± 11 GPa and 1181 ± 71 MPa, respectively.

As a summary, unidirectional alignment of rCFs is crucial in achieving high-performance rCF-based composites. Strategic positioning of the fibres is key to maximising the composite's mechanical characteristics by carrying loads and stresses in the right directions. However, as described in the previous paragraphs, it is challenging to achieve unidirectional alignment of rCFs in conventional applications. The next section explores feasibility of achieving unidirectional alignment in advanced manufacturing methods.

### Advanced Manufacturing Methods to Enhance the Performance of rCF Composites

Advanced manufacturing methods include Additive Manufacturing (AM) [139], Automated Tape Laying (ATL) [98] and Automated Fibre Placement (AFP) [140]. The section investigates how advanced manufacturing methods strengthen rCF applications by addressing current limitations while achieving desired composite performance by precise control of the processing direction of semi-product tapes and yarns during composite manufacturing. The placement and orientation of the semi product within the composite is influenced by advanced manufacturing methods such as automated tape or fibre placement and filament winding method but not the alignment or orientation of the individual carbon fibres in the semi product itself. For instance, the AFP system employs robotic arms to accurately place continuous strands of semi-long and long length rCF staple, tape, or yarn while aligning them spatially and spatially orienting the ply. Though carbon fibre alignment within these semi products is left unchanged. Furthermore, these technologies enable fibre architectures to be customized to meet specific applications requirements [141]. Additive manufacturing provides the precise control of material deposition necessary to efficiently utilize rCFs, improving part performance, and permitting tailoring of the reinforcement strategy. One particularly beneficial use of this is for reclaimed semi long/long fibres from wastes to be able to be integrated into complex geometries, thus minimizing waste and maximizing resource efficiency. The transformational potential of additive manufacturing arises from the ability to address challenges encountered by traditional manufacturing with semi-long and long fibres. One advantage of an AM process vs. conventional is that AM enables strategic placement and orientation of fibres (for example) inside a part, thereby maximising strength and stiffness in the areas where they are most needed. The addition of rCFs can be adapted to the advanced AM techniques of directed energy deposition and fused deposition moulding (FDM/FFF). Moreover, AM offers the possibility for producing multi material designs, thereby producing hybrid structures which incorporate rCFs with virgin fibres or other reinforcements leading to lightweight, high-performance composites. The broadened adoption of AM for semi long/long rCFs has the potential to revolutionize composite manufacturing by creating tailored design, less material waste, and greater sustainability.

**Fig. 12:**
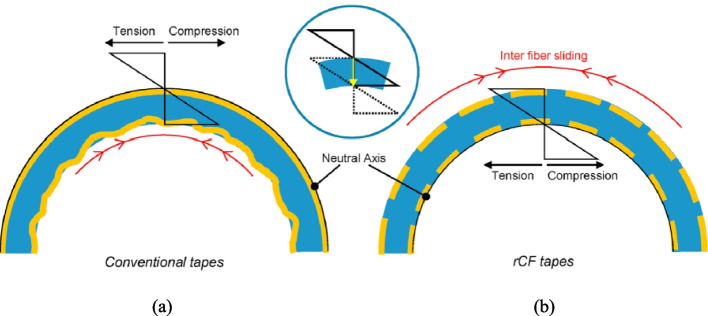
Curved tape placement using continuous CFRP and discontinuous semi-long/long rCFRP [132]

Semi-long/long rCF are highly formable in applications in curved products. This unique feature can make composites using semi-long/long rCFs maintain or even improve the mechanical and structural performances [132, 142]. As illustrated in Fig. 12(a), when continuous and virgin CFs are used for fabricating curved products, the CFs on the outside radius cannot extend, resulting in a neutral layer of fibres in which the fibres are not compressed or tensioned and hence are wasted. While the CFs on the inside radius become compressed, resulting in a reduction in their mechanical properties [132]. In contrast, as shown in Fig. 12(b), using semi-long and long length rCF based filament or tapes will prevent the above mentioned problems from occurring [98, 143]. Furthermore, with precision fibre placement in AM, semi-long and long rCFs can be strategically positioned to increase the overall strength and stiffness of a composite product. Advanced shaping and moulding techniques enhance this process, resulting in highly aligned semi-long rCF-based mats and tapes that are easily drawable and formable.

ATL is well suited for rCF applications, particularly in the automotive industry. In ATL, continuous strips of semi-long and long length rCF-based tape are precisely applied to moulds to minimize material waste and optimize mechanical properties. With ATL, the production processes are automated to ensure consistent quality and optimum efficiency, making it an ideal tool for the remanufacturing of semi-long/long rCF components.

AFP technology can play an important role in the remanufacturing of semi-long/long rCFs for renewable energy applications, such as wind turbine blades. The AFP system uses robotic arms to lay down continuous strands of semi-long and long length rCFs staples of fibre or yarns while precisely controlling fibres orientations and the ply angles. A uniform and proper distribution of rCFs is ensured through this method, enhancing the structural integrity and performance of wind turbine blades.

## Conclusions

This review paper has provided a thorough analysis of the current techniques for recycling, preparation, remanufacturing, and application of semi-long (25–100 mm) /long (> 100 mm) rCFs. It is found that using semi-long/long rCFs manufacture composites of high performance is possible, but it is important to select the most appropriate techniques at different stages. The main findings of this review are as follows:

- Mechanical recycling methods are only advantageous in recovering fibrous recyclates. Whilst chemical and electrochemical recycling methods are more suitable for semi-long/long rCFs. However, the most promising recycling method is the Electrically Driven Heterocatalytic Decomposition (EHD).
- Surface coating or sizing rCFs remains essential to improve rCF-based composite performance by solving issues of fibre residuals and weak interfacial bonding strength between fibre and matrix. Proper selection of sizing materials and techniques according to the application ensure uniform fibre treatment and enhance the overall durability and performance of composite.
- Prior to further processing, it is necessary to open and separate semi-long/long rCFs, regardless of the recycling techniques. The most prevalent method is carding, which can improve the uniformity of the fibres, making them more suitable for further processing.
- To manufacture semi-long/long rCFs into randomly aligned mats and tapes, air-laid, wet-laid, and carding methods can be utilized; among them, roller carding is the most effective method for producing randomly aligned mats of uniform thickness and high fibre volume fraction.
- It is imperative to achieve unidirectional fibre alignment and to increase the fibre volume fraction ratio in composites made from semi-long/long rCFs because only unidirectional alignment of rCFs can achieve high-performance composites. Among all remanufacturing methods for rCFs, friction spinning stands out due to its ability to highly align and process semi-long/long rCFs.
- Using advanced manufacturing processes including additive manufacturing and automated fibre placement allows more precise alignment and customization of fibres for specific high-performance applications. These methods can also improve repeatability, reduce variability, minimize material waste, and handle complex designs in a more efficient way.

## Data Availability

No datasets were generated or analysed during the current study.
